# Irreversible left-ventricular lead electrical failure from conductor externalization managed with left bundle branch area pacing: a case report

**DOI:** 10.1093/ehjcr/ytag047

**Published:** 2026-01-25

**Authors:** Jaber Almohammad, Ahmed Almarzuqi, Habib Rehman Khan

**Affiliations:** London Health Sciences Centre Research Institute, Western University, 339 Windermere Road, London, ON N6A 5A5, Canada; London Health Sciences Centre Research Institute, Western University, 339 Windermere Road, London, ON N6A 5A5, Canada; London Health Sciences Centre Research Institute, Western University, 339 Windermere Road, London, ON N6A 5A5, Canada

**Keywords:** Left-ventricular lead, Conductor externalization, Coronary-sinus lead, Conduction system pacing, Left bundle branch area pacing, Case report

## Abstract

**Background:**

Conductor externalization is a recognized mechanism of transvenous lead failure. In left-ventricular (LV) coronary-sinus (CS) leads, irreversible electrical failure requiring extraction is uncommon. Conduction system pacing with left bundle branch area pacing (LBBAP) can provide a physiological alternative when CS re-implantation is not feasible.

**Case summary:**

A 67-year-old man with non-ischaemic cardiomyopathy and reduced LV ejection fraction (EF) secondary to infective endocarditis with severe aortic/mitral regurgitation underwent bioprosthetic aortic valve replacement with mitral repair and cardiac resynchronization therapy-defibrillator implantation in 2021 after drug-associated torsade de pointes and ventricular fibrillation arrest. He underwent transcatheter edge-to-edge mitral repair in June 2024. At routine review in October 2024, he was clinically well and asymptomatic; LV lead testing showed very high impedance (>3000 Ω), threshold 5.75 V at 1.0 ms, and intermittent loss of capture. Chest radiography showed stable lead position. At revision in March 2025, the extracted LV lead displayed conductor externalization, and CS re-implantation was precluded by a small, unwireable anterolateral branch. LBBAP was implanted with low capture threshold and stable sensing; the generator was replaced without complications. At 1-month follow-up, the LV EF was 20–25% (previously 10–15% in 2024); by August 2025 he was New York Heart Association (NYHA) class I and euvolaemic with stable weight.

**Conclusion:**

This case demonstrates persistent electrical failure in LV CS lead with extraction-confirmed conductor externalization, contrasts with prior reports of electrically silent or transient disturbance, and supports LBBAP as a practical physiological option when CS re-implantation is not feasible.

Learning points

- Persistent LV lead impedance elevation with intermittent loss of capture should raise suspicion for conductor externalization and lead failure.
- When CS re-implantation is not feasible, LBBAP can deliver physiological resynchronization with low thresholds and stable performance.

## Introduction

Lead-related mechanical and electrical problems remain an important cause of cardiac resynchronization therapy (CRT) failure. Conductor externalization through an insulation breach is well described in implantable cardioverter-defibrillator leads such as Riata, whereas reports in LV coronary-sinus (CS) leads are uncommon; when present, they may be mechanically evident yet electrically silent.^1–4^ When CS re-implantation is not feasible, conduction system pacing (CSP) with left bundle branch area pacing (LBBAP) provides a physiological alternative route to resynchronization.^5,6^ We report a QuickFlex LV lead with conductor externalization and persistent electrical failure requiring extraction, followed by successful LBBAP after failed CS re-implantation.

## Summary figure

**Table:** 

Date	Event	Key details
2021	AVR + MV repair; CRT-D implantation	Bioprosthetic AVR with mitral repair after infective endocarditis; CRT-D placed for torsades/VF (drug-associated long QT).
Jun 2024	Transcatheter edge-to-edge mitral repair	MitraClip performed for severe MR.
21 Oct 2024	Routine device interrogation	LV threshold 5.75 V @ 1.0 ms; impedance 1150→>3000 Ω; intermittent loss of capture; BiV pacing >99%.
22 Oct 2024	Chest radiography	Stable device/lead positions.
Mar 2025	Revision procedure	LV CS lead extracted; conductor externalization confirmed. CS re-implantation precluded (small, unwireable anterolateral branch). LBBAP implanted; generator replaced.
Apr 2025	Echocardiography	LVEF 20–25% (was 10–15% in 2024).
Aug 2025	Heart-failure clinic	NYHA class I; euvolaemic; weight ∼84–86 kg; paced rhythm.

Abbreviations: AVR, aortic valve replacement; MV, mitral valve; CRT-D, cardiac resynchronization therapy defibrillator; MR, mitral regurgitation; LV, left-ventricle/ventricular; CS, coronary-sinus; LBBAP, left bundle branch area pacing; BiV, biventricular; NYHA, New York Heart Association; LVEF, left-ventricular ejection fraction.

## Case presentation

The patient had non-ischaemic cardiomyopathy considered secondary to infective endocarditis (IE) with severe aortic and mitral regurgitation. He underwent bioprosthetic aortic valve replacement with mitral valve repair in 2021. A CRT-D was implanted in 2021 after drug-associated QT prolongation from amiodarone and antiretroviral therapy with torsades de pointes and cardiac arrest from ventricular fibrillation (VF). Prior to routine device assessment in 2024, he had several admissions with heart-failure exacerbations. He underwent transcatheter edge-to-edge mitral repair in June 2024 for severe mitral regurgitation.

**Figure 1:**
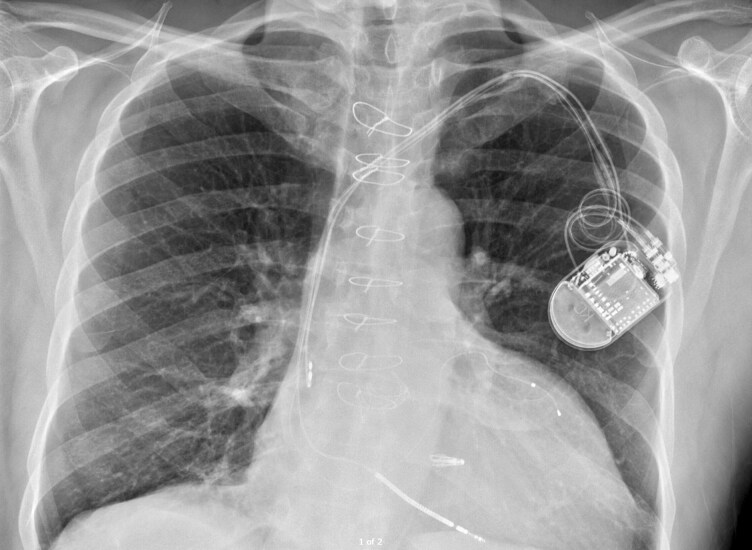
A chest radiograph in October 2024 showed stable generator and lead positions, making macro-dislodgement unlikely.

At routine device review on 21 October 2024, he was clinically well and asymptomatic, reporting no chest pain, palpitations, dyspnoea, or peripheral oedema. Interrogation demonstrated intermittent loss of LV capture despite high outputs, with LV threshold up to 5.75 V at 1.0 ms and impedance >3000 Ω; biventricular pacing remained >99%. A chest radiograph on 22 October 2024 showed stable generator position and lead courses, with no radiographic evidence of macro-dislodgement (*Figure 1*). Empiric anticoagulation was not initiated given no independent indication and no clinical evidence of thromboembolism. As he was clinically stable and travelling, a remote transmission was planned before 5 November 2024 and revision was scheduled for March–April 2025.

**Figure 2:**
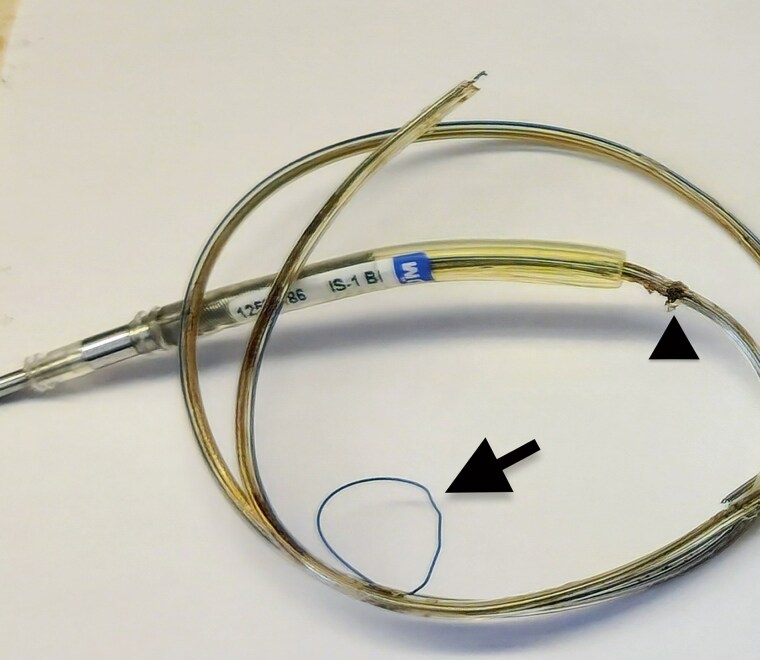
Extracted LV CS lead demonstrating conductor externalization (arrow) and the probable fracture site (arrowhead).

On 31 March 2025, under local anaesthesia and conscious sedation, the pocket was reopened and the LV CS lead was wired with a Whisper guidewire and mechanically extracted. A SafeSheath 10.5 Fr could not be advanced beneath the clavicle, so new left axillary venous access was obtained. Using a deflectable sheath, the CS was cannulated; occlusive venography revealed a small-calibre, unwireable anterolateral branch, precluding CS re-implantation. LBBAP was then performed using a SelectSecure™ 3830 lead (Medtronic) advanced to the left septum, achieving a qR in V1 and a paced QRS duration of 180 ms. Acute parameters were: threshold 0.6 V at 0.5 ms, impedance 460 Ω, R wave 14 mV, stimulus-to-peak V6 84 ms, and V6–V1 40 ms. A CRT-D (Medtronic, model DTMA2D4) was connected and implanted in a TYRX antibacterial pouch. Programming included DDD 80–105 beats per minute. Tachy therapy zones were: Monitor VT 400 ms; fast VT 320 ms (active); VF 240 ms (active). There were no acute complications. Total fluoroscopy time was 27.1 min; entrance dose 193 mGy; dose–area product 1938.8 cGy·cm^2^. The extracted lead body showed conductor externalization through an insulation breach (*Figure 2*. Replacement of the generator mitigated prior concerns about battery drain from sustained high LV output.

**Diagnostic reasoning.** Differential considerations for abrupt very high impedance with capture loss included macro-dislodgement, header or set-screw issue, vector-specific dysfunction, conductor fracture, and insulation breach with conductor externalization. Macro-dislodgement was unlikely given the stable chest radiograph and >99% biventricular pacing. Header or set-screw problems were unlikely given stable atrial and right-ventricular lead performance and reproducible LV abnormalities. Extraction confirmed conductor externalization, explaining the open-circuit behaviour.

**Follow-up.** Echocardiography on 17 April 2025 showed LV ejection fraction (EF) 20–25%. At Heart-Failure Clinic on 18 August 2025, he was NYHA class I and euvolaemic, with paced rhythm on the electrocardiogram and stable weight ∼84–86 kg. Serial weights rose from ∼74 kg in June 2024 to ∼86 kg by March 2025, then stabilized after LBBAP implantation. Blood cultures in February 2024 showed no growth.

**Pathophysiology.** Conductor externalization is thought to result from chronic mechanical stress at the distal silicone-insulated segment, producing inside-out insulation abrasion and eventual conductor protrusion.^1–4^ The electrical signature is a shift to markedly elevated, often fluctuating impedance with rising capture thresholds and intermittent or absent capture.^1–3^ Imaging may suggest an irregular lead course, but definitive confirmation is by direct visualization at extraction.^1–3^

## Discussion

Reports of LV CS lead externalization frequently describe no measurable electrical consequence or only transient disturbance.^1–4^ In contrast, this case demonstrated persistent, irreversible electrical failure with very high LV impedance and loss of capture that could not be corrected by reprogramming, together with direct visualization of an externalized conductor on the explanted lead.^1–4^ These findings underscore surveillance considerations for LV CS leads with suspected externalization: sustained increases in LV impedance and capture thresholds with intermittent or absent capture should prompt device-indicated revision rather than reliance on symptoms alone.^1–4^ Because the externalized segment lay intravascularly, there was theoretical concern for infection in a patient with previous IE; blood cultures were obtained and were negative, and there were no clinical features of device infection.

**Figure 3:**
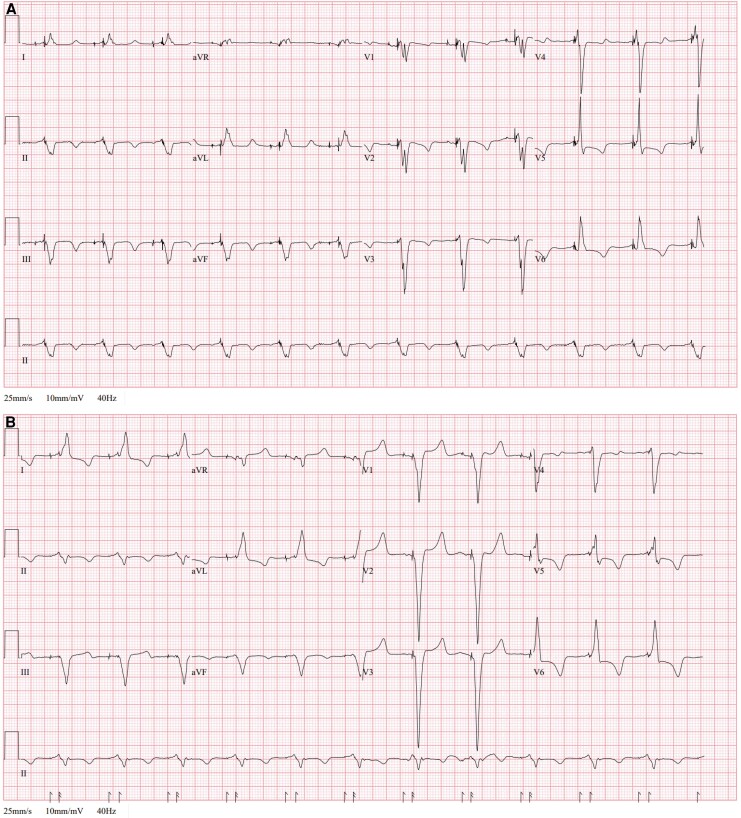
Twelve-lead ECGs at 25 mm/s and 10 mm/mV. Panel A: biventricular pacing with the LV CS QuickFlex lead in 2024 (QRS 200 ms). Panel B: left bundle branch area pacing in 2025 (QRS 180 ms).

CS re-implantation was anatomically precluded by a small, unwireable anterolateral tributary. LBBAP therefore provided a physiological route to resynchronization, consistent with evidence that CSP can substitute for CS CRT when venous anatomy or prior lead failure limit options.^5,6^ Electrical performance was favourable with low capture thresholds and stable sensing; paced QRS narrowed from 200 ms (biventricular pacing) to 180 ms with LBBAP (*Figure 3*), consistent with improved electrical synchrony. This restored durable pacing at low output and provided a viable resynchronization strategy when CS anatomy constrained lead placement.^5,6^

## Conclusion

Irreversible LV lead electrical failure with conductor externalization may present with persistently elevated impedance and loss of capture. When CS re-implantation is precluded, LBBAP can provide effective physiological resynchronization with favourable electrical performance and clinical stability.

## Lead author biography


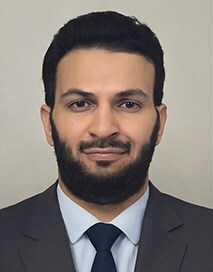


Dr Jaber Almohammad, MD, is a cardiology resident at Western University, London, Ontario, Canada. He has a strong interest in cardiac electrophysiology, particularly in arrhythmia management, device therapy, and pacing strategies. He is actively engaged in clinical and case-based research with the goal of improving patient care and advancing knowledge in cardiovascular medicine.

## Data Availability

All clinical details supporting this report are contained within the article.

## Lead author statement

The lead author affirms that this manuscript is an honest, accurate, and transparent account; no important aspects have been omitted.

## References

[1] Hauser RG, McGriff D, Retel LK. Riata implantable cardioverter-defibrillator lead failure: analysis of explanted leads with a unique insulation defect. Heart Rhythm 2012 May;9:742–749. 22209723 10.1016/j.hrthm.2011.12.019

[2] Theuns DAMJ, Elvan A, De Voogt W, De Cock CC, Van Erven L, Meine M. Prevalence and presentation of externalized conductors and electrical abnormalities in Riata defibrillator leads after fluoroscopic screening: report from the Netherlands Heart Rhythm Association Device Advisory Committee. Circ Arrhythm Electrophysiol 2012 Dec;5:1059–1063. 23091049 10.1161/CIRCEP.112.975755

[3] Mendenhall GS, Saba S. Electrical dysfunction associated with conductor externalization of a silicone left ventricular lead. Pacing Clin Electrophysiol 2015 Mar 1;38:357–361. 25582957 10.1111/pace.12563

[4] Jude S, Meyer AJ. St. Jude Medical Issues Physician Communication about QuickSite and QuickFlex LV CRT Leads ProQuest document link FULL TEXT [Internet]. 2012. https://www.cardiovascular.abbott/content/dam/cv/cardiovascular/pdf/reports/St_Jude_Medical_Issues_Physician_Communication_about_QuickSite_and_Quic.pdf

[5] Hua J, Chen Y, Yu J, Xiong Q, Xia Z, Xia Z, et al Long-term outcomes of left bundle branch area pacing versus biventricular pacing in patients with heart failure and complete left bundle branch block. Heart Vessels 2022 Jul 1;37:1162–1174. 35088204 10.1007/s00380-021-02016-5 PMC9142423

[6] Diaz JC, Duque M, Aristizabal J, Marin J, Niño C, Bastidas O, et al The emerging role of left bundle branch area pacing for cardiac resynchronisation therapy. Arrhythm Electrophysiol Rev 2023;12:e29. 38173800 10.15420/aer.2023.15 PMC10762674

